# Efficient Peptide-Mediated In Vitro Delivery of Cas9 RNP

**DOI:** 10.3390/pharmaceutics13060878

**Published:** 2021-06-14

**Authors:** Oskar Gustafsson, Julia Rädler, Samantha Roudi, Tõnis Lehto, Mattias Hällbrink, Taavi Lehto, Dhanu Gupta, Samir EL Andaloussi, Joel Z. Nordin

**Affiliations:** 1 Centre for Biomolecular and Cellular Medicine, Department of Laboratory Medicine, Karolinska Institutet, 141 57 Stockholm, Sweden; oskar.gustafsson@ki.se (O.G.); julia.radler@ki.se (J.R.); samantha.roudi@ki.se (S.R.); mattias.hallbrink@ki.se (M.H.); taavi.lehto@ki.se (T.L.); dhanu.gupta@ki.se (D.G.); samir.el-andaloussi@ki.se (S.E.A.); 2 Institute of Technology, University of Tartu, Nooruse 1, 50411 Tartu, Estonia; tonis.lehto@ut.ee

**Keywords:** cell-penetrating peptide (CPP), CRISPR/Cas9, RNP, drug delivery, PepFect14, gene editing, non-viral, nanoparticle

## Abstract

The toolbox for genetic engineering has quickly evolved from CRISPR/Cas9 to a myriad of different gene editors, each with promising properties and enormous clinical potential. However, a major challenge remains: delivering the CRISPR machinery to the nucleus of recipient cells in a nontoxic and efficient manner. In this article, we repurpose an RNA-delivering cell-penetrating peptide, PepFect14 (PF14), to deliver Cas9 ribonucleoprotein (RNP). The RNP-CPP complex achieved high editing rates, e.g., up to 80% in HEK293T cells, while being active at low nanomolar ranges without any apparent signs of toxicity. The editing efficiency was similar to or better compared to the commercially available reagents RNAiMAX and CRISPRMax. The efficiency was thoroughly evaluated in reporter cells and wild-type cells by restriction enzyme digest and next-generation sequencing. Furthermore, the CPP-Cas9-RNP complexes were demonstrated to withstand storage at different conditions, including freeze-thaw cycles and freeze-drying, without a loss in editing efficiency. This CPP-based delivery strategy complements existing technologies and further opens up new opportunities for Cas9 RNP delivery, which can likely be extended to other gene editors in the future.

## 1. Introduction

With the discovery of clustered regularly interspaced short palindromic repeat (CRISPR)-associated 9 (Cas9) there has been an explosion of research and output within the gene-editing field making it the most widely researched gene-editing tool both in vitro and in vivo to date [1]. There is also a vast therapeutic interest in the use of Cas9. The protein has not only proved itself to be highly specific and simple to use but has furthermore been utilized in combination with a plethora of different fusion proteins, where novel functions have been added to Cas9 [1]. These functions include, but are not limited to, gene regulation and de novo gene DNA repair pathways [2,3,4,5]. Cas9 has enabled a new beginning for gene editing, and while Cas9 may not be the final protein chosen for the mainstream treatment of a genetic disorder, the protein chosen is likely to be a CRISPR derivative. Several clinical studies are being performed or have been completed utilizing CRISPR/Cas9 to cure genetic disorders [6]. However, this is a challenging task since the delivery of the endonuclease machinery needed for genome editing is difficult [7]. This difficulty arises due to the large size and complexity of the gene-editing machinery. Many of the human trials implement ex vivo delivery of Cas9 to hematopoietic stem cells using electroporation [8,9]. Furthermore, in in vivo trials, viruses and/or lipids have been evaluated as delivery vehicles; however, significant toxicity has been reported with the use of lipids and viruses [10,11].

Adeno-associated viruses (AAVs) have been used successfully for Cas9 delivery in several animal models. However, AAVs suffer from drawbacks such as limited packaging size, pre-existing immunity to the AAV-vector, possible detrimental long-term expression, as well as a higher risk of integration of the delivered DNA template after Cas9-mediated DNA double-stranded breaks (DSB) [12,13,14]. Cas9 is commonly delivered in DNA or RNA format, however, these come with their own disadvantages, such as prolonged expression leading to increased off-target cleavage risk, DNA integration into the genome, and immune responses against excessive amounts of Cas9, as reviewed by Doudna [7]. This explains why the majority of trials done in humans so far have used ex vivo delivery, thus enabling rigorous quality control before returning the edited cells to the patient. Recently, one such trial aiming to treat Sickle cell anemia and β-Thalassemia was reported, where the erythroid-specific enhancer region of BCL11A was targeted by CRISPR/Cas9 in CD34^+^ hematological stem cells ex vivo. The editing efficiency in the cells was then analyzed before being returned to the patients [6]. The delivery of Cas9 in mRNA format reduces the risks involved but retains the temporal complexity since the guide RNA (gRNA) must be present and intact by the time Cas9 has been translated into protein.

Cas9 can additionally be delivered as a protein complexed with gRNA. Delivering Cas9 as a ribonucleoprotein (RNP) has several advantages, such as lower and shorter presence inside of the target cells, which leads to decreased risk for off-target cleavage and immune reactions. Moreover, it solves the temporal challenge of Cas9 and gRNA being required to be present in the same cell at the same time [15]. However, delivering Cas9 as an RNP is challenging since Cas9 is a large protein with no uniform charge, while the RNP has an overall negative charge imparted from the gRNA which enables delivery methods relying on charge interactions [15,16]. Altogether this means that there is an unmet need for novel delivery methods for Cas9-RNPs to minimize the unwanted effects of Cas9 treatment. The most commonly used delivery methods today for Cas9-RNPs are electroporation and lipid nanoparticles, where the cationic lipids can interact with the anionic RNP to form particles capable of achieving therapeutic levels of editing in mice [8,9,17].

The lack of viable, non-lipid, in vivo RNP delivery methods is not something that has gone unnoticed. There is a flurry of new methods being developed for Cas9-RNP delivery in vitro, likely with the hope for further in vivo development. Examples of these are gold nanoparticles, induced transduction by osmocytosis and propanebetaine (iTOP), Exosomes, Nanoclew DNA nanoparticles, and many others [18,19,20,21].

Another promising delivery method utilizes cell-penetrating peptides (CPPs) to deliver the RNPs. CPPs are generally short, 10–30 amino acids (aa) in length, with a cationic charge, and can be further divided based on their hydrophobic and amphipathic properties [22]. These CPPs have successfully been used to deliver oligonucleotides, proteins, and small molecule drugs [23]. The delivery of cargo using CPPs is commonly achieved through the direct coupling of the CPP to the cargo. The delivery of proteins using CPPs in a non-covalent fashion was reported as early as 2001 but has lately gained more traction [24]. The CPP-mediated delivery of Cas9 RNP has been explored before, however, previous attempts at RNP delivery using CPPs required large amounts of Cas9 and achieved low editing rates resulting in reduced therapeutic potential [10,25,26]. Furthermore, CPPs are often positively charged, meaning that any Cas9-CPP fusion will contain regions of high positive charge, possibly interfering with Cas9 complexation to the gRNA [27]. Thus, a non-covalent approach to RNP-CPP delivery is likely needed to achieve efficient editing. This has been done by Montenegro et al., who are, to the best of our knowledge, the first group to publish non-covalent supramolecular delivery of Cas9 RNP using a CPP screen to identify CPPs for functional RNP delivery [28].

We have previously co-reported on the development of a synthetic CPP, PepFect14 (PF14), that was shown to be capable of the highly efficient delivery of different short splice-switching oligos (SSOs) in a non-covalent manner [29]. PF14 is an amphipathic, N-terminally stearylated CPP that was based on the previous generation, stearyl-transportan10 peptide vector, where the lysines and isoleucines were exchanged for ornithines and leucines [30]. We hypothesized that PF14 could be repurposed to deliver Cas9 RNPs by non-covalent interaction with the gRNA component of the Cas9 RNP. PF14 can form nanoparticles with SSOs in different buffers by simple incubation at room temperature (RT). If this could be extended to Cas9 RNP, it would represent a very simple and efficient transfection method with the potential for translation to an in vivo setting.

## 2. Materials & Methods

Cas9 protein, CRISPR RNA (crRNA), tracr RNA (trRNA), single-guide RNA (sgRNA), and oligonucleotides were purchased from IDT (Integrated DNA Technologies, Coralville, IA, USA). The oligos used and their sequences can be found in Table 1. The gRNA contained IDT standard modifications. The PepFect14, Stearyl-AGYLLGKLLOOLAAAALOOLL-NH_2_, was purchased from Pepscan Preseto and dissolved in water before use. The antibodies used can be found in Table 2.

PVA-PEG (polyethylene glycol–polyvinyl alcohol, i.e., Kollicoat^®^ IR), HEPES, glucose, and sucrose (Merck, Sweden). Lipofectamine™ (RNAiMAX), Lipofectamine™ CRISPRMAX™ (cat#13778075, cat# CMAX00008, Thermo Fisher Scientific, Waltham, MA, USA).

### 2.1. Cell Culture

HEK293T Stop-Light (SL), MDA-MB-231 (MDA) SL, and MCF-7 (MCF) SL were all kindly provided by Dr. Olivier de Jong, HEK293T WT were obtained from ATCC. The SL cells are stably transduced, monoclonal, cells that express the SL construct; these cells have been used for Cas9 editing screens in previously published work [31]. All cells were cultured and maintained in Dulbecco’s modified Eagle’s medium (DMEM^®^) GlutaMax™, Gibco™ containing 10% FBS (Invitrogen) together with 1% Penicillin/Streptomycin (100 U/mL and 100 mg/mL) in a humidified incubator, at 37 °C with 5% CO_2_. The cells were regularly split using 0.05% trypsin (Invitrogen).

### 2.2. Cas9 RNP Preparation

Cas9 was complexed with either crRNA/trRNA (cr/trRNA) or sgRNA according to IDTs instructions for Cas9 RNP formation. New complexes were prepared before each experiment. cr/trRNA and sgRNA form a highly similar structure that interacts with the Cas9 protein, both activating and guiding Cas9.

### 2.3. Protein to Peptide Complexation

Antibody: for the PepFect14-mediated delivery of antibodies, a molecular ratio (MR) of 1:50 was used as determined as optimal in initial experiments (data not shown). The antibody was added to the HBG buffer (HEPES-buffered glucose solution; 20 mM HEPES, 5% glucose, pH = 7.2) which had been mixed 1:1 with a PVA-PEG solution (2.5 *w/v*% PVA-PEG and 6.3 *w/v*% sucrose dissolved in H_2_O). PF14 was added to the AB/buffer solution, which was vortexed immediately. The mixture was incubated at RT for 30 min in low-binding tubes before addition to the cells.

Cas9 RNP: RNPs were added either to HBG, HBG mixed 1:1 with a 10% PVA-PEG (10 *w/v*% PVA-PEG and 6.3 *w/v*% sucrose dissolved in H_2_O) solution, or DMEM mixed 1:1 with a 10% PVA-PEG solution, depending on the experiment. PF14 was added to the RNP/buffer solution, which was vortexed immediately.

For HEK293T transfections, a DMEM/PVA-PEG solution with a final PVA-PEG *w/v*% of 5 was used, unless otherwise stated.

For MDA- and MCF-cells, an HBG/PVA-PEG solution with a final PVA-PEG *w/v*% of 1.5 was used.

PF14 was added in different MRs, Cas9 RNP to PF14 (RNP: PF14) (Cas9 conc. was constant at a final concentration of 10 ng/µL during complexation). The mixture was incubated at RT for 40 min in low-binding tubes. This was followed by the addition of the Cas9-PF14 complexes to cells growing in full media in 96-well plates. Depending on the total amount of Cas9 desired per well, varying volumes of Cas9-RNP complexes were added to the wells and incubated for 3 days before analysis.

### 2.4. RNAiMAX and CRISPRMax RNP Transfection

RNAiMAX transfection was performed following the protocol optimized by Chesnut et al., while CRISPRMax transfection was carried out according to the manufacturer’s instructions [32]. The transfections were done in full media (10% FBS), with the lipofectamine RNP complexes incubated with the cells for 3 days.

### 2.5. Flow Cytometry

For antibody delivery, HEK293T WT cells were seeded one day before treatment (3 × 10^4^ cells/well) in a 96-well plate. The cells were then treated with AB-PF14 prepared as described above, incubated in full media for 1 h followed by PBS wash, and singularized using trypsin-EDTA (0.05%) (Thermo Fisher Scientific). The cells were stained with DAPI (0.1 µg/mL) (Sigma) before analysis by flow cytometry on a MACSQuant Analyzer 10 instrument equipped with three lasers (405, 488, 638 nm; Miltenyi Biotec, Bergisch Gladbach, Germany). PE was detected in channel B2 (585/40 nm), APC in R1 (655–730 nm), FITC in B1 (525/50 nm), and DAPI in channel V1 (450/50 nm). Events were gated for cells/single cells/viable cells/(PE or APC or FITC) positive cells (Supplementary Materials Figure S1). Data were analyzed using FlowJo 10.6.2 (FlowJo, LLC) software.

For Cas9 RNP gene-editing, cells were seeded one day before treatment (10^4^ cells/well) in a 96-well plate. The cells were then treated with RNP-PF14 and incubated in full media without a media change for 3 days to enable robust eGFP expression. The cells were prepared for flow cytometry as described above for AB-treated cells. eGFP was detected in channel B1 (525/50 nm), mCherry (mC) in B3 (655–730 nm), and DAPI in channel V1 (450/50 nm). A minimum of 2000 cells per well were analyzed. The cells were gated in the order of cells/viable/mC^+/^eGFP^+^. (Supplementary Materials Figure S3). Data were analyzed using FlowJo 10.6.2 (FlowJo, LLC) software.

### 2.6. DLS

The size and zeta potential of the complexes were determined by dynamic light scattering using a Zetasizer Nano ZS apparatus (Malvern Instruments). Complexes were formulated as described above in 50 μL volume and measured for size. Prior to the zeta potential measurement complexes were diluted 16 times with 10 mM HEPES buffer at pH 7.4.

Measurements were carried out at 22 °C. For each size measurement, the number of runs was set to 5 (run duration 10 s) and number of measurements was set to 3. For zeta potential the number of measurements was set to 3 but measurement number and duration were set to automatic. The experiments were repeated 3 times and mean values of each measurements were generated in Malvern software and the averages of the 3 mean values are presented with SEM.

### 2.7. Microscopy

Cas9 RNP-PF14 were complexed and added to the cells growing in full media as previously described and incubated for 3 days, whereupon the cells were imaged using a fluorescence microscope (Olympus IX81, Olympus America Inc. Center Valley, PA, USA). Automatic settings for exposure and contrast were used to visualize each treatment.

### 2.8. Storage Testing

For SpeedVac, 50 µL of the formed complexes were added to an Eppendorf tube and until dry in a Thermo Scientific Savant SC210A SpeedVac under a vacuum without added heating. Samples were resuspended in 50 µL H_2_O and added to cells as normal.

A similar process was used for freeze-drying where 50 µL of the samples were frozen at −80 °C, transferred into a cooling block, which in turn had been chilled to −20 °C, before being placed in a vacuum until dry. Similarly to above, the lyophilized samples were resuspended in 50 µL H_2_O and used as normal. The freeze-thawing was done by placing the samples in –80 °C and then thawing at 37 °C, which was repeated the desired number of times.

### 2.9. DNA Extraction

Genomic DNA was extracted using Sera-Mag™ Carboxylate-Modified Magnetic Particles (Thermo Fisher Scientific). Briefly, cells were first incubated with an SDS lysis buffer (1% SDS, 50 mM Tris-HCl, 10mM EDTA) containing Proteinase K (QIAGEN, Cat No./ID: 19131), followed by the addition of RNAse A (Fisher Scientific, FEREN0531). The cell lysate was then incubated with Sera-Mag beads for 5 min, followed by 3 EtOH washing steps while placed on a magnet. DNA was eluted in buffer EB (QIAGEN, Cat. No./ID: 19086).

### 2.10. PCR and DNA Analysis Methods

The SL construct’s target region was PCR-amplified using HotStarTaq Master Mix Kit (Qiagen). The PCR reaction was carried out under the following conditions: 95 °C for 15 min, 37 cycles of 94 °C for 40 sec, 54 °C for 1 min, and 72 °C for 1 min, and one cycle at 72 °C for 10 min. 5 µL of the PCR product was then digested with 0.5 µL Thermo Scientific FastDigest XhoI enzyme in a total reaction volume of 15 µL, followed by gel electrophoresis on a 2% agarose gel in TAE buffer. The gel was visualized on a Molecular Imager^®^ VersaDoc™ MP 4000 system, and the band’s intensities were quantified using Image Lab software (Bio-rad, Hercules, CA, USA). The percentage of indels was calculated as ((a/(a + b + c)) × 100), where a is a band percentage of the undigested PCR product and b and c are the band percentages of the digested product.

### 2.11. Statistical Analysis

All statistical analysis was done by GraphPad Prism (8.3.0), the analysis methods used are described in the relevant figure legends. All error bars are SEM.

### 2.12. Next-Generation Sequencing (NGS)

Gene editing analysis was performed using targeted next generation sequencing through TIGERQ AB, Lund, Sweden. Briefly, total genomic DNA was isolated from the target cells, and the target editing site was amplified by PCR (2kb). PCR products were subject to NexteraXT fragmentation and paired-end (2 × 150 bp) Illumina sequencing. An analysis of results files was carried out with specialized gene editing analysis to call variant sequences and their respective modification frequencies.

## 3. Results

### 3.1. Cas9 RNP Delivery

**Figure 1:**
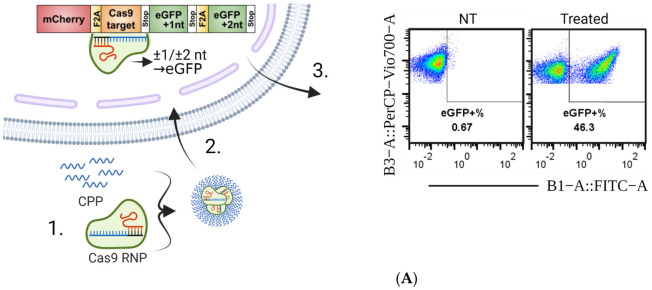

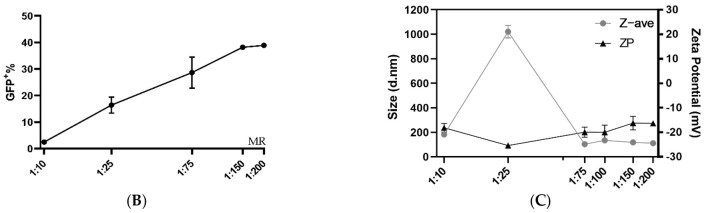
Overview of the experimental setup and PF14 delivery of Cas9 RNP to reporter cells. (**A**) Outline of the general experimental setup. 1. PF14 is complexed with Cas9 RNP and added to cells. 2. The PF14 enables RNP to escape into the cytoplasm, where the nuclear localization signal relocates the RNP to the nucleus, where it cleaves the DNA in the SL construct. 3. The cells are incubated for 72 h after transfection and then analyzed by flow cytometry. (**B**) Flow cytometry results from the HEK293T SL cells treated with the 200 ng Cas9 RNP complexed with PF14 in increasing MR (RNP: PF14). The complexes were formed in HBG buffer. *n* = 3. (**C**) Size and zeta potential of the HBG-formulated RNP: PF14 complexes in increasing MR, as analyzed by DLS.

To investigate the possibility of protein delivery using PF14, fluorescently labelled antibodies (ABs) conjugated to either FITC, APC, or PE were complexed with PF14 and added to HEK293T WT cells. The cells incubated with PF14-AB showed a large increase in mean fluorescent intensity and percentage positive cells over the naked AB, Supplementary Materials Figure S2A,B, the signal increase correlated with the size of the attached fluorescent tag. With the encouraging data from AB delivery by PF14, we next aimed to evaluate the delivery of Cas9-RNPs by PF14. To this end, the delivery of Cas9 RNPs was investigated by utilizing a reporter system where sequence-specific editing leads to eGFP expression [31]. The SL reporter constructs constitutively express mC and co-express functional eGFP if indel formation from the Cas9 induced DSB causes a frameshift, which in turn can be analyzed by flow cytometry (see a schematic representation of the system in Figure 1A). Hence, the percentage of eGFP^+^ cells equals the percentage of edited cells. The strategy used for the gating in the flow cytometry analyses is shown in Supplementary Materials Figure S3. The cells were previously generated and extensively sorted by collaborators, to obtain a pure population as reported in a previous publication [31]. The constitutively expressed mC allows for a simple evaluation of construct expression level in the reporter cells. This set-up allowed us to effectively screen RNP–CPP complexation conditions in a 96-well format (see Figure 1A for a schematic figure of the experimental workflow with the reporter cells).

To test if PF14 can deliver Cas9 RNPs, RNPs were diluted in HBG buffer, followed by the addition of PF14 in increasing MRs to the Cas9 RNP. The Cas9 added to cells was kept constant at 200 ng Cas9 protein, thus the final concentration in the wells was 8.9 nM Cas9 RNP. To our surprise, the complexation of Cas9 RNP with PF14 was sufficient to achieve editing in full media (10% FBS in DMEM) at higher MRs (Figure 1B). Thus, the formed nanoparticles are stable enough to endure the destabilizing effects of the serum in the media, enabling non-covalent protein delivery to cells. According to the DLS measurements, the formed nanoparticles appear to have a stable size, at around 100 nm (Supplementary Materials Figure S4), and negative zeta-potential at higher RNP to PF14 molar ratios (Figure 1C).

### 3.2. PVA-PEG Addition to Complexation Increases Editing Rates

**Figure 2:**
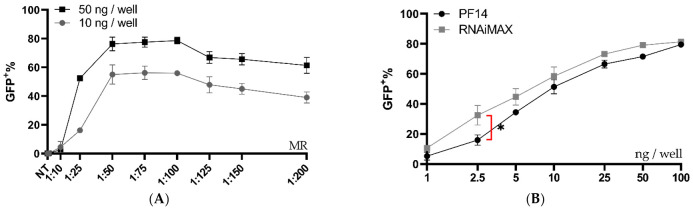
Ratio and dose testing of Cas9 RNP complexed with PF14 in a DMEM/PVA-PEG buffer. (**A**) Ratio testing, RNP: PF14, using 10 and 50 ng Cas9 (0.61 or 2.8 nM Cas9) per well in HEK293T SL cells. *n* = 3 for 10 ng, *n* = 2 for 50 ng. There was no statistical difference (*p* > 0.05) of MR between 1:50 to 1:200 for the 10 ng dose as analysed by analysis of variance (ANOVA), followed by Tukey’s post-hoc test. (**B**) Dose titration of RNP-PF14 (MR 1:100 RNP: PF14) plotted together with RNAiMAX. Doses ranging from 1 ng (0.06 nM) to 100 ng (5.18 nM) per well of HEK293T SL cells. Linear *y*-axis against log^10^ *x*-axis. There was a significant difference (*, *p* = 0.0101) between RNAiMAX and PF14 at 2.5 ng per well, the remaining doses showed no significant difference, as analysed by two-way ANOVA followed by Bonferroni’s multiple comparison test, *n* = 3.

Although the PF14 delivery of functional Cas9-RNPs was encouraging, we believed the editing rates could be further improved by optimizing the formulation conditions. Thus, another formulation buffer was investigated using a similar set-up as in Figure 1B. Previous studies have reported that the addition of poly (vinyl alcohol) (PVA) or polyvinylpyrrolidone (PVP) to dendrimer oligo transfections increases transfection efficiency, while polyethylene glycol (PEG) is a very commonly used additive in the medical field [33]. PVA-PEG is related to PVA and PVP and is a commonly used additive in a wide range of industries, including in the food industry. We hypothesized that the addition of PVA-PEG could increase the local concentration of RNP and CPP and thus enhance their complexation. With this novel buffer set-up, the optimal MR between Cas9 RNP and PF14 was again investigated, however with a decreased Cas9 dose per well (50 ng per well, which corresponds to 2.8 nM Cas9). After the addition of PVA-PEG, the editing rates reached approximately 80%, which is likely the maximum eGFP+ positive cells for this assay, since editing leading to +/− 3 bases would not lead to eGFP expression (Figure 2A). Hence, the experiment was repeated with a reduced dose of 10 ng Cas9 per well (corresponding to 0.61 nM Cas9). With the lower dose, no significant difference was observed in editing between MRs in the range of 1:50 to 1:200, although the results indicate that MRs higher than 1:100 are not beneficial; therefore 1:100 was chosen due to its low variation and as adding more peptide moreover increases the risk for toxicity (Figure 2A). Attempts to characterize the particles formed in the presence of PVA-PEG were made (Supplementary Materials Figure S4). However, the large amount of PVA-PEG-derived particles most likely obscured the RNP: PF14 particles, thus, the physico-chemical characterization of these complexes was not possible by DLS. Next, a dose titration was performed using an MR of 1:100 in the PVA-PEG buffer which resulted in a dose-dependent increase of gene editing, starting at the lowest dose tested, 1 ng Cas9 (corresponding to 0.06 nM Cas9-RNP). At 10 ng (0.61 nM), approximately 50% of the cells were successfully edited, while approximately 80% of the cells were edited at 50 ng (2.8 nM) and 100 ng (5.18 nM) (Figure 2B). Furthermore, the editing levels achieved with PF14 are comparable to RNAiMAX (Figure 2B). However, RNAiMAX is an extensively optimized product and has been further optimized for Cas9 RNP delivery by Chesnut et al., thus the results with PF14 were impressive given the early stages of its testing [32]. RNAiMAX was used due to it being more reliable in our hands than CRISPRMax, while still retaining very similar editing levels (Supplementary Materials Figure S5).

### 3.3. Validation of the Editing Efficiency

**Figure 3:**
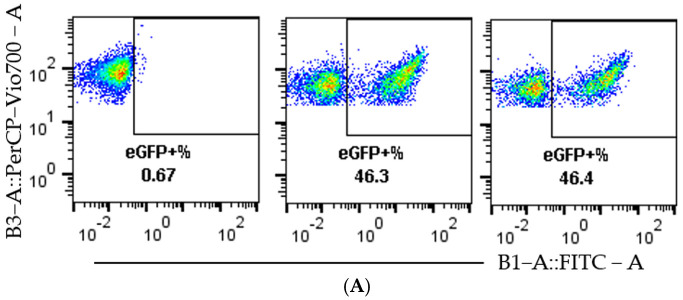

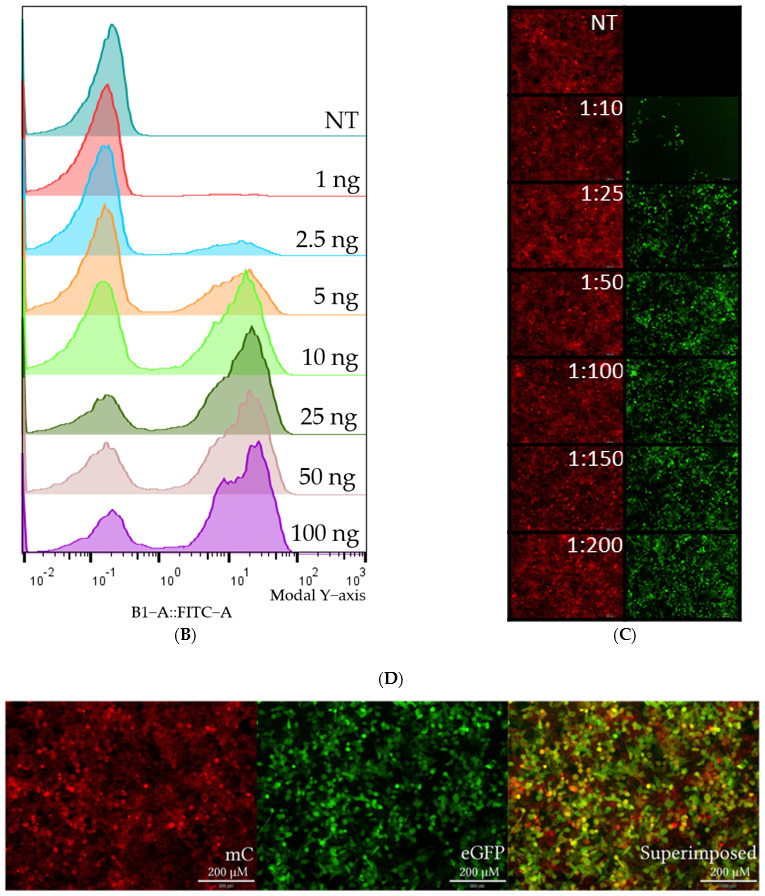
Visualization of representative flow cytometry data and microscopy. HEK293T SL cells were used for Figure 3A–D. (**A**) Dot plots of mC (*y*-axis) plotted against eGFP (*x*-axis). In the order left to right, non-treated, 10 ng Cas9 RNP-PF14 (MR 1:100, RNP: PF14), and RNAiMAX 10 ng Cas9 RNP. (**B**) Histogram visualizing the eGFP signal in RNP-PF14 treated cells, added text displays Cas9 (ng)/well. (**C**,**D**) Fluorescence microscopy (10×) displaying the mC and eGFP of cells treated with Cas9 RNP-PF14, 50 ng/well in an increasing MR ratio (**C**) or 100 ng Cas9/well (MR 1:100) (**D**).

A more detailed analysis of the flow cytometry data, seen in Figure 3A, shows a clear separation between non-edited cells, mC^+^ eGFP^−^, and edited cells, mC^+^ eGFP^+^, which act as an indication of the quality of the reporter model. This separation remains clear over the tested dose range, as can be seen in Figure 3B. To confirm the function of the reporter system beyond flow cytometry, fluorescence microscopy was performed on cells treated with RNP-PF14 (Figure 3C,D), which corroborated the flow cytometry results and showed viable cells at all MRs.

**Figure 4:**
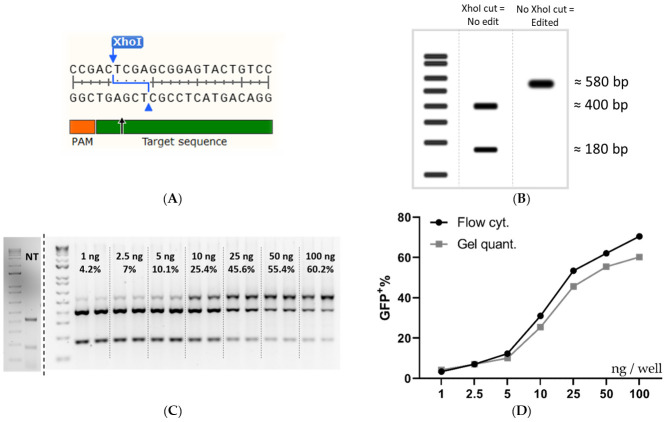
Validation of the flow cytometry data by RFLP analysis. (**A**) The DNA sequence at the Cas9 cleavage site in SL cells. (**B**) Expected RFLP outcome at 0 and 100% XhoI disruption. (**C**) An agarose gel was used for intensity quantification for RFLP analysis of HEK293T SL cells treated with increasing amounts of RNP: PF14 (MR 1:100). The dose of Cas9 per well (ng) and the mean quantified editing is superimposed on the gel image. (**D**) Comparison of a dose titration between flow cytometry quantification and RFLP quantification. Linear *y*-axis against log^10^ *x*-axis. Flow cytometry quantification was done in triplicate, *n* = 1. Gel quantification was done in duplicate, *n* = 1. The cells in (**C**,**D**) were HEK293T SL cells treated with an increasing dose of Cas9/well (MR 1:100 RNP: PF14, formulated in DMEM/PVA-PEG (5 *w/v*%)).

Next, the flow cytometry data was further validated by using restriction fragment length polymorphism (RFLP) and NGS analysis. The experiment done in Figure 2B was repeated and analyzed by flow cytometry, once again, followed by DNA extraction and genetic analysis by RFLP and NGS of selected samples. The Cas9 cut-site in the SL reporter construct coincides with an XhoI restriction enzyme (RE) cleavage site, which was utilized to evaluate indel formation since any indel formation in the XhoI site would change the sequence and abolish the RE sequence (Figure 4A). Figure 4B depicts the expected outcome of gel electrophoresis after XhoI cleavage with no editing and complete Cas9 editing. The RFLP assay showed a dose-dependent increase replicating the flow cytometric data (Figure 4C), generated both in Figure 2B and Figure 4D. The quantification of the gel bands yielded an editing rate similar to, but slightly lower than the one recorded by flow cytometry (Figure 4D). The data shown in Figure 4D are from the same wells and can thus be directly compared.

**Figure 5:**
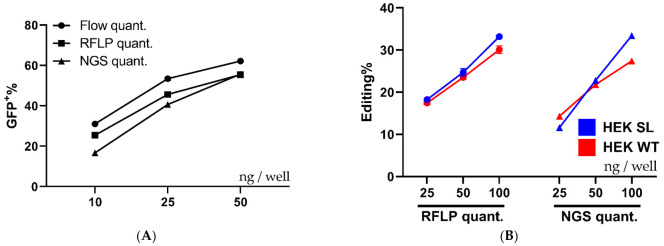
Comparison of quantification techniques and cell types. (**A**) Shows a comparison between different quantification techniques, flow cytometry, RFLP, and NGS performed on the same samples of HEK293T SL cells treated with increasing amounts of RNP: PF14 complexes (MR 1:100 RNP to PF14). *n* = 1, with the flow cytometry done in triplicate, gel quant in duplicate, and NGS done in single samples. Part of the cells were analyzed by flow cytometry, the remainder of the cells were analyzed by RFLP and NGS after DNA purification and PCR amplification. (**B**) Displays the quantified editing of HPRT in HEK293T SL and HEK293T WT cells treated with increasing amounts of RNP: PF14 complexes (MR 1:100 RNP to PF14). The gRNA used targets the human HPRT gene. The quantification by RFLP and NGS was performed on the same samples. Gel quantification was performed in duplicate (*n* = 1), while NGS was done a single time per treatment (*n* = 1).

Quantification by RFLP is not always 100% accurate, therefore NGS was carried out on the same DNA extracted for gel quantification. The editing rates reported by flow cytometry, RFLP, and NGS were surprisingly similar, although, the flow data may overestimate the editing slightly. That being said, the samples follow the same trend regardless of the analysis method, and hence the flow cytometric readout can be utilized for screening purposes without further confirmation (Figure 5A).

To further evaluate PF14 for Cas9-RNP delivery the human HPRT gene in HEK293T SL cells and HEK293T WT cells was targeted to validate functional delivery beyond the reporter cell assay. The human HPRT gRNA target sequence contains a BfaI RE site, thus an RFLP assay can be utilized similarly to the XhoI RE for the SL target sequence. As seen in Figure 5B, the editing of HPRT in SL cells and WT cells occurs at similar levels, as quantified by RFLP and NGS. It appears that the HPRT sgRNA is less efficient than the SL sgRNA, since the HRPT sgRNA achieved around 30% editing at 100 ng Cas9 per well, while the SL sgRNA achieved over 50% using half the Cas9 amount, as quantified by NGS. The editing outcomes as quantified by NGS from the treatments in Figure 5A,B can be seen in Supplementary Materials Figure S6, where the proportion of each outcome is quantified using NGS. The majority of indels are deletions, independently of gRNA target and cell type.

**Figure 6:**
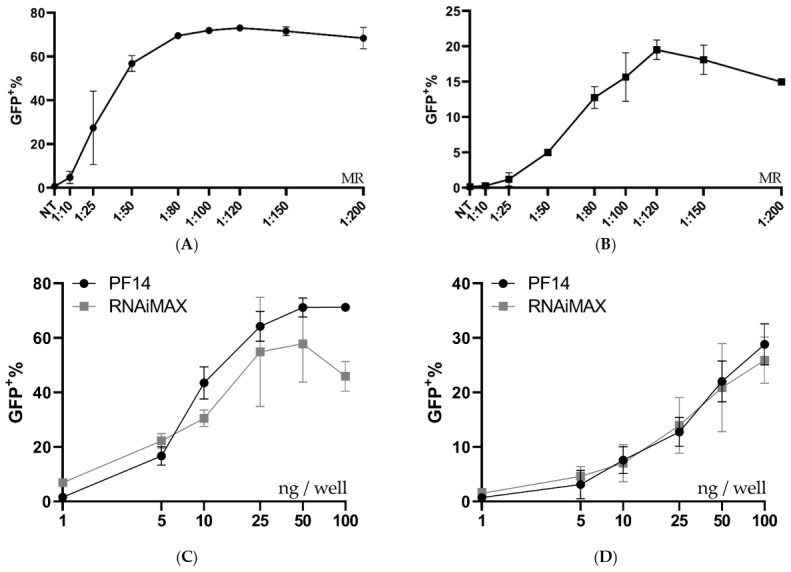
Ratio and dose testing in MDA-MB-231 SL and MCF-7 SL. (**A**,**B**) Ratio optimization of Cas9 RNP-PF14 (50 ng Cas9/well, formulated in DMEM/PVA-PEG (2.5 *w/v*%)) given to MDA SL and MCF SL cells. Means of *n* = 2. (**C**,**D**) Dose titration of RNP-PF14 (MR 1:150 RNP to PF14 formulated in DMEM/PVA-PEG (2.5 *w/v*%)) and RNAiMAX on (**C**) MDA SL and (**D**) MCF SL cells, respectively. Linear *y*-axis against log^10^ *x*-axis. *n* = 3 for PF14-RNP and *n* = 2 for RNAiMAX.

To investigate if the PF14 delivery capabilities can be extended to other cell types, MDA SL and MCF SL cells were treated with RNP-PF14 complexes, which had been complexed in DMEM/PVA-PEG. Both cell lines are breast cancer-derived cells that are generally more refractory to transfection compared to HEK293T cells. Different MRs were evaluated in both cell types, with the best editing rates again observed around MR 1:100 (Figure 6A,B). When comparing the peptide to RNAiMAX in MDA SL and MCF SL cells, the peptide worked on par with or better than RNAiMAX, especially in the MDA SL cells (Figure 6C,D).

### 3.4. Storage Conditions Effect on Transfection Efficiency

**Figure 7:**
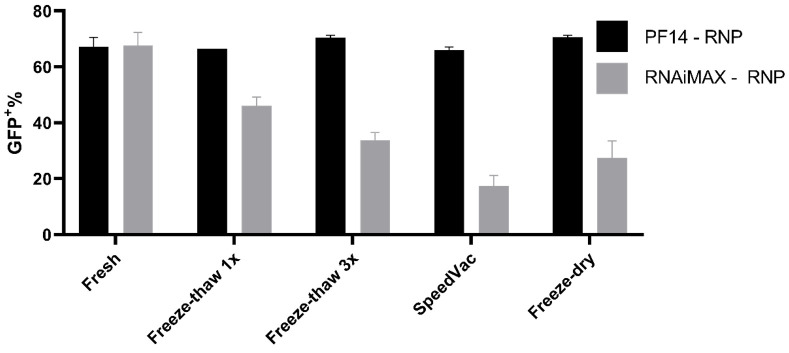
Storage capabilities of PF14-Cas9 RNP complexes. Comparison of 50 ng Cas9 complexed with either PF14 (MR 1:150) or RNAiMAX and subsequent editing efficiency after different storage conditions. The Cas9 RNP-PF14 was formulated in DMEM/PVA-PEG buffer. *n* = 2.

PF14 complexes can withstand various storage conditions, thus the storage properties of our Cas9 RNP-PF14 nanoparticles were tested [29]. The RNP-PF14 nanoparticles were first complexed as previously outlined and then frozen for freeze-thawing experiments, freeze-dried, or vacuum concentrated (SpeedVac) without being frozen. The formed nanoparticles not only survived freeze-thaw cycles, SpeedVac, and freeze-drying, but they were equally active after storage (Figure 7. This enables easy handling and usage. RNAiMAX on the other hand has a reduced transfection ability after any of the storage techniques tested.

## 4. Discussion

The therapeutic capabilities of Cas9 and its fusion proteins are rapidly increasing, however, the ability to deliver these therapeutics to cells is behind the pace at which new therapeutics are being developed. The most successful RNP delivery methods to date have been based on lipoplex delivery due to their simplicity and effectiveness. However, lipofectamine-type agents have drawbacks, such as toxicity at higher or repeated doses [10]. A promising alternative delivery method explored in this manuscript is the use of CPPs as the delivery vehicle. One of the first CPPs developed in the 80s was the HIV-derived trans-activator of transcription (TAT) peptide, which was shown to be able to deliver nucleotides and proteins [34]. Developments and iterations have yielded peptides with impressive delivery capabilities; despite this, publications describing Cas9 RNP delivery using peptides are rare [10,26]. Many of the previous Cas9-CPP publications rely on the direct attachment of the CPP to Cas9 and suffer from surprisingly low editing efficiency, requiring repeated treatments using Cas9 concentrations in the µM range [10,26].

In this work, we showed that PF14, originally developed by the Langel lab for nucleotide delivery, can be repurposed for the delivery of Cas9 RNP in a non-covalent manner. The ease of manufacturing and efficiency of editing shows that the CPP delivery of complex proteins such as Cas9 RNP is a viable approach. The concept of non-covalent protein delivery using CPPs was first published in 2001 when Morris et al. used Pep-1 to deliver proteins in a non-covalent fashion [27]. Additionally, Montenegro et al. are to our knowledge the first group to publish the non-covalent supramolecular delivery of Cas9 RNP using a CPP screen to identify CPPs for functional RNP delivery [24]. Their RNP-CPP treatment achieved 38% editing of HPRT after the addition of 160 nM RNP and required a media change after 4 h of incubation, likely due to toxicity. In comparison, our RNP-PF14 achieved 33% in HPRT after the addition of only 5.2 nM without the need for a media exchange, which is a reduction in dose by around 30 times. However, Montenegro et al. used Hela cells, while in this manuscript HEK293T cells were utilized. Additionally, the guide sequences were different, these two factors mean that a direct comparison is not possible. Nevertheless, the editing efficiencies by PF14-mediated delivery were further corroborated at low doses in both MDA and MCF cells. The importance of the editing results in these cells is significant since HEK cells are notoriously easy to transfect, thus efficient editing in other cell types is of great translational value.

This work using PF14 to deliver Cas9 RNP achieved editing levels that are, to our knowledge, one of the most efficient examples of Cas9 RNP delivery using a non-lipoplex delivery approach, achieving over 50% editing with a Cas9 concentration of 0.61 nM. It moreover shows the possibility of repurposing non-covalent nucleotide delivering CPPs for the delivery of Cas9 RNPs.

The editing efficiency is lower without the added PVA-PEG. The addition of PVA-PEG to nanoparticle formulations has not been published before, thus this tool could be useful for related research into nanoparticle formation and pinpoints how important the buffer composition is for successful complexation and delivery. However, when formulated in HBG buffer, the editing efficiency was 38.9% in HEK293T SL cells at 8.9 nM Cas9-RNP, which is still high.

The internalization pathways utilized by PF14-RNP were not investigated in this work, but we hypothesize them to be similar to the pathways utilized by PF14-oligonucleotide complexes which were extensively investigated by Ezzat et al. [35,36]. Ezzat et al. could determine that the uptake of PF14-oligonucleotide complexes was mediated by class-A scavenger receptors. Furthermore, it is generally accepted that most CPPs are endocytosed after binding to proteoglycans on the surface of cells, thus, PF14-RNPs are likely endocytosed after either binding scavenger receptors or proteoglycans on the cell surface [37,38,39,40].

The SL reporter system utilized allowed for extensive screening in a 96-well format for the optimal conditions of RNP delivery. However, reporter systems do not always correlate with editing levels in an endogenous system; thus, the editing results were validated on a genetic level in the reporter system using RFLP and NGS. Both analyses corroborated the editing levels detected using flow cytometry, although the true editing percent appears to be marginally lower than that observed by flow cytometry. This could be due to the possible incorporation of several SL sequences in the reporter cell genome, thus leading to a high eGFP^+^ percent with a slightly lower editing percentage on the genetic level due to the Cas9 cleaving only one of several SL sites in that particular cell. If this is the case, then each eGFP^+^ cell would still represent a successful delivery of Cas9 RNP to that cell. The insert copy number of the SL reporter system has not been thoroughly investigated on a genetic level. However, multiple inserts are expected to yield several populations of varying eGFP intensity after RNP delivery, which is something not seen in the eGFP flow cytometry histograms (Figure 3B). Multiple SL construct insertions into the reporter cells would likely lead to a higher percentage of eGFP^+^ cells due to increased Cas9 activity arising from an increased amount of Cas9 targets. However, as mentioned above, each eGFP^+^ cell would still represent a successful nuclear Cas9 delivery event. The SL system has a theoretical maximum eGFP percentage output since only indels of 1 or 2 nucleotides lead to eGFP expression. Logically, one could assume the maximum of the system is 66%, since two-thirds of indels lead to a frameshift of 1 or 2 nucleotides. However, previous publications have shown that the indel distribution is biased towards certain indels depending on the genomic context around the DSB [28]. Scaffidi et al. examined indel profiles in over 1000 sites in the genome after Cas9 transfection and found that 81% of the detected indels lead to a frameshift, hence, for the reporter system to have a maximum read-out of around 80% is not surprising [28]. However, Scaffidi et al. transduced sgRNA into Cas9-expressing HepG2 cells, thus the conditions were not identical.

To test if the reporter cassette changed the transfection efficiency, HEK SL and HEK WT were transfected with Cas9 RNP-PF14 targeting HPRT. The lack of difference between the two indicates that the uptake and release of CPP nanoparticles are equal between the two. This in turn acts as a validation of the reporter system.

Lastly, the complexes formed are highly resilient to storage conditions, showing no reduction in transfection efficiency after freeze-thawing, SpeedVac, or freeze-drying. This is not surprising, since the Langel lab has previously reported that PF14 complexed with SSOs retain 100% activity after SpeedVac drying [29]. However, it is surprising and promising that the Cas9 RNP, which is made up of a protein and RNA, survived the storage conditions when formulated with PF14. In this study we have used RNA guides that comprise chemically modified nucleotides at the ends of the gRNA, hence the artificial gRNA is likely more stable than a native gRNA and can explain the enhanced stability observed during the storage study.

The delivery of Cas9 has been touted as a game-changer in the gene therapy field and while this is true, Cas9 is intrinsically limited to treating only genetic disorders arising from aberrant protein expression. This makes up only a small subset of genetic diseases, as most diseases are caused by a loss of function, rather than a gain of function, arising from indels and substitutions which require precise editing. This is something that Cas9 alone cannot achieve due to the dominant, non-precise repair mechanism of the Cas9-induced DSB, non-homologous end-joining. Several Cas9 fusions have been developed to overcome this limitation, arguably the most known among them are the base- and prime-editing fusions developed by the Lui lab [2,3,4,5]. These proteins are significantly larger than the native Cas9, meaning that they cannot be packaged into a single AAV particle. We believe that our methodology is well situated to deliver larger fusion proteins, based on the results from the antibody delivery shown in Supplementary Materials Figure S2 where the AB with the largest tag, PE (240 kDa), and a total molecular weight close to 500 kDa, had the highest fold-increase in fluorescence over antibody alone. Hence the size of the protein does not appear to be the limiting factor for delivery to cells by PF14.

In conclusion, this report shows the potential of the non-covalent delivery of Cas9 RNP. It also highlights that CPPs developed for other purposes can be repurposed for the delivery of ribonucleoproteins such as Cas9. The successful delivery of gene editors as RNPs is expected to be important for the future of gene editing, thus it is of great importance to further develop delivery methods for Cas9 RNPs. We believe that CPPs comprise excellent potential and can be further optimized for effective Cas9 delivery.

## Figures and Tables

**Table 1:** Oligonucleotides used and their sequences.

**sgRNAs**	**Target Sequence**
Stop-Light guide RNA (gRNA)	GGACAGTACTCCGCTCGAGT
HPRT (Hypoxanthine-guanine phosphoribosyltransferase) Human gRNA	AATTATGGGGATTACTAGGA
**PCR primers**	**Sequence**
PCR primer-Stop-Light FWD	ACATCACCTCCCACAACGAG
PCR primer-Stop-Light REV	GGTCTTGTAGTTGCCGTCGT
PCR primer-HPRT FWD	AAGAATGTTGTGATAAAAGGTGATGCT
PCR primer-HPRT REV	ACACATCCATGGGACTTCTGCCTC

**Table 2:** Antibodies used, fluorescent tags, and relevant physio-chemical information.

Target	Tag	Tag Weight (kDa)	Tag Isoelectric Point	Purchased from Biosciences.
Rat IgG2a kappa Isotype Control (eBR2a)	FITC	0.39	4.70	cat# 554680
CD45.1 Monoclonal Antibody	APC	105	5.00	cat# 17045382
Mouse IgG1, κ Isotype Control	PE	240	4.15	cat# 554680

## Data Availability

Raw data can be shared upon request by contacting the corresponding author.

## Supplementary Materials

The following are available online at https://www.mdpi.com/article/10.3390/pharmaceutics13060878/s1, Figure S1 Shows the gating of a representative PF14-AB(PE) treated sample. Figure S2: MFI Fold change and uptake percentage of AB-PF14 compared to naked AB. Figure S3 Shows the gating of a representative PF14-RNP – 10 ng Cas9, DMEM/PVA-PEG, treated HEK293T SL sample. Figure S4: Size (A) and Zeta-potential (B) of RNP-PF14 complexes in HBG and PVA-PEG buffer or just PVA-PEG buffer. Figure S5: Editing efficiencies of RNAiMAX and CRISPRMax, two lipofectamine positive controls for RNP delivery. Figure S6 shows the distribution of indel variants in SL and WT cells treated with an increasing amount of RNP-PF14 targeting the SL construct or the HPRT gene.
