# Supplementary material for: Efficient Peptide-Mediated In Vitro Delivery of Cas9 RNP
Source: Pharmaceutics. 2021 Jun 14;13(6):878. doi: 10.3390/pharmaceutics13060878 (PMC8232299; doi:10.3390/pharmaceutics13060878)
Supplement: Supplementary file 1 [file pharmaceutics-13-00878-s001.zip › pharmaceutics-1202856-SI.pdf]

pharmaceutics

MDPI

Supplementary Materials: Efficient Peptide-Mediated In Vitro Delivery of Cas9 RNP

Oskar Gustafsson, Julia Rädler, Samantha Roudi, Tõnis Lehto, Mattias Hällbrink, Taavi Lehto, Dhanu Gupta, Samir EL Andaloussi and Joel Z Nordin

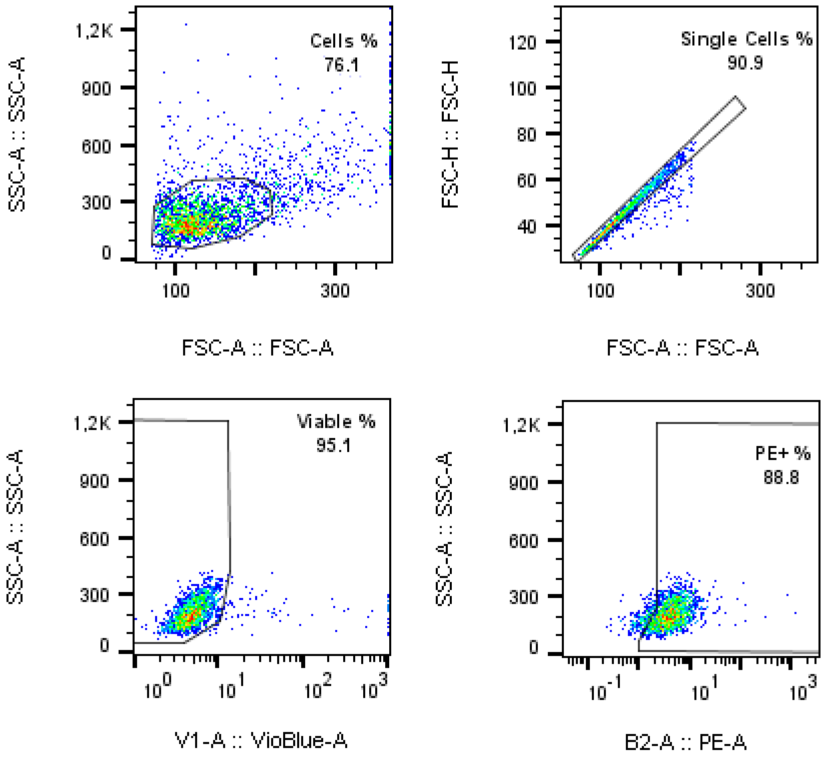

Figure S1. Shows the gating of a representative PF14-AB(PE) treated sample. The cells were gated in the order of Cells / Single / Viable / PE or APC or FITC Data were analyzed using the FlowJo 10.6.2 (FlowJo, LLC) software.

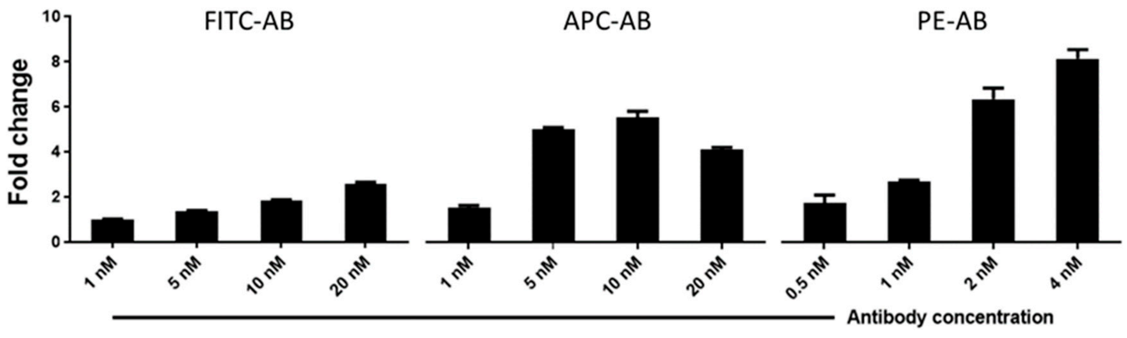

(**A**)

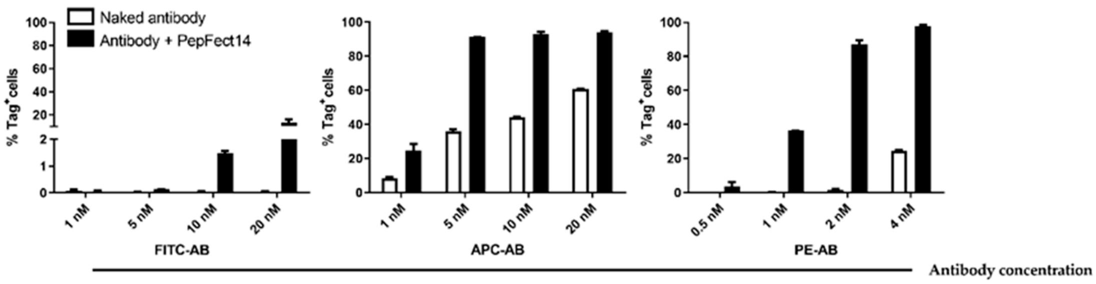

(**B**)

Figure S2. MFI Fold change and uptake percentage of AB-PF14 compared to naked AB. (**A**) Shows the fold change of MFI of PF14 complexed AB over the naked AB control given at the same concentration. $n = 1$. (**B**) Displays the percentage of cells positive for the used fluorescent tag. The PE-AB was used in a lower concentration due to it quickly reaching 100% positive cells, thus, higher amounts would make comparisons impossible.

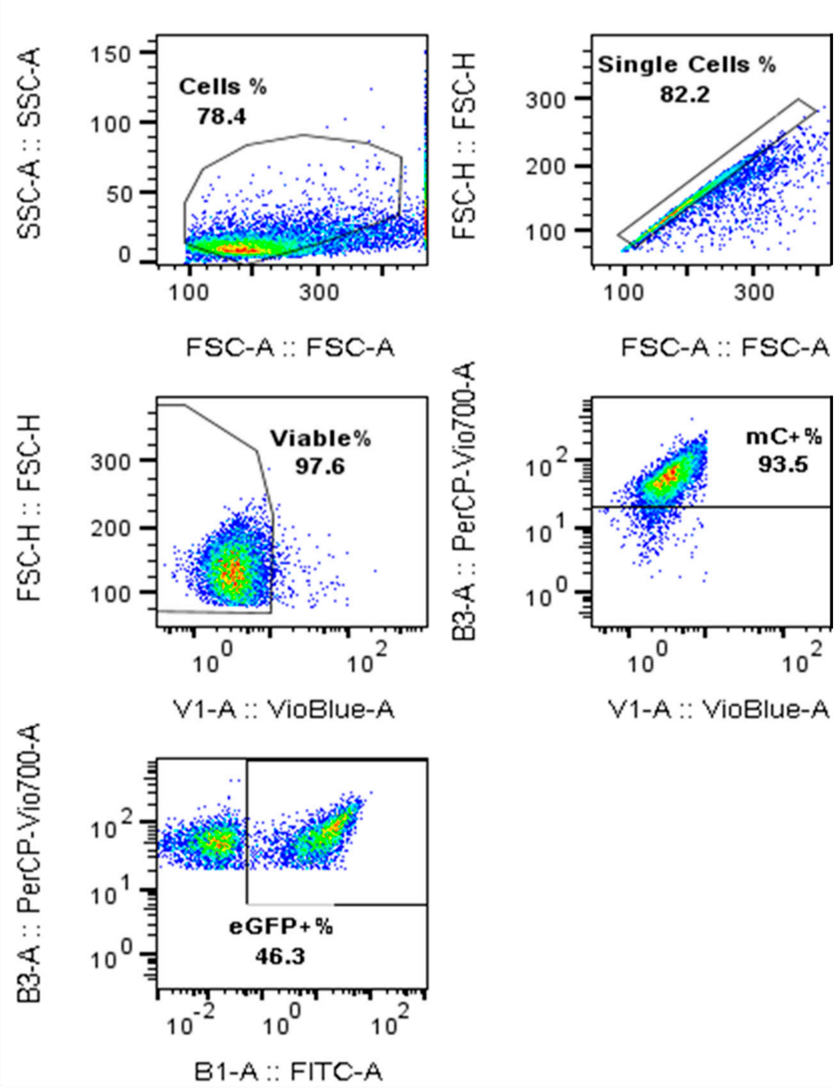

Figure S3. Shows the gating of a representative PF14-RNP – 10 ng Cas9, DMEM/PVA-PEG, treated HEK293T SL sample. The cells were gated in the order of Cells / Single cells / Viable / mC^{+} / $eGFP^{+}$. Data were analyzed using the FlowJo 10.6.2 (FlowJo, LLC) software.

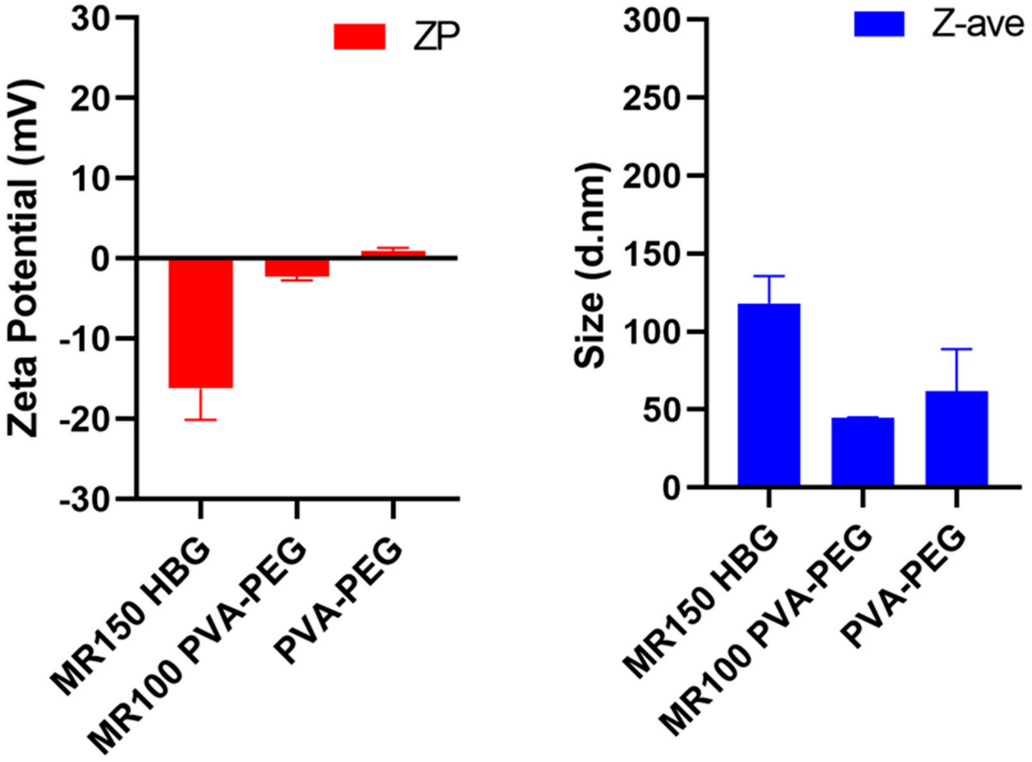

(A) **(B)**

Figure S4. Size (**A**) and Zeta-potential (**B**) of RNP-PF14 complexes in HBG and PVA-PEG buffer or just PVA-PEG buffer.

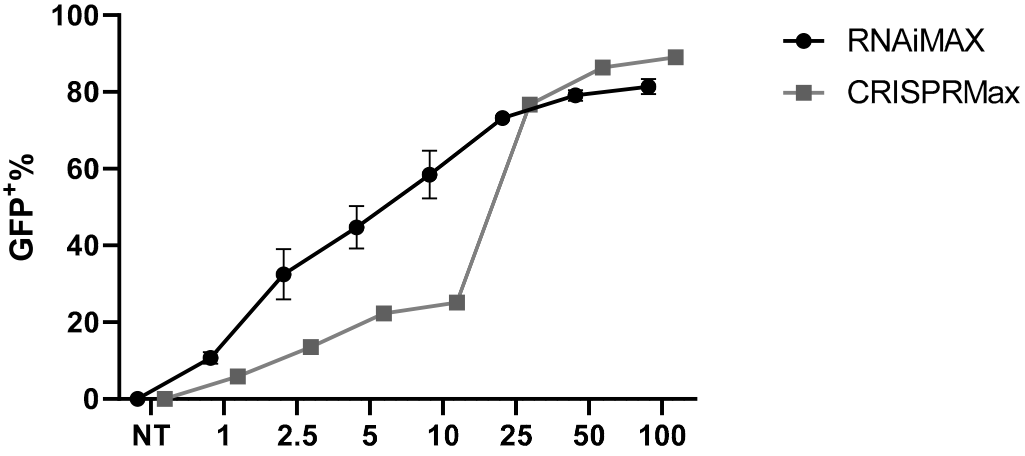

Figure S5. Editing efficiencies of RNAiMAX and CRISPRMax, two lipofectamine positive controls for RNP delivery. $n = 3$ for RNAiMAX, $n = 1$ for CRISPRMax.

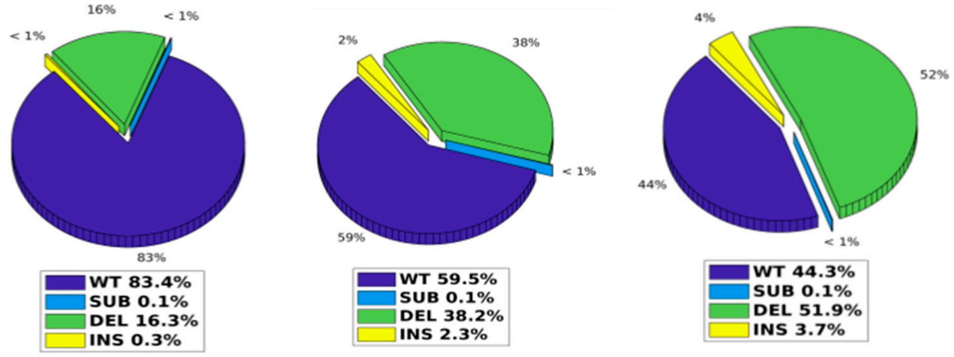

(**A**) Stop-light HEK293T cells – Stop-light gRNA – 10, 25, and 100 ng Cas9/well

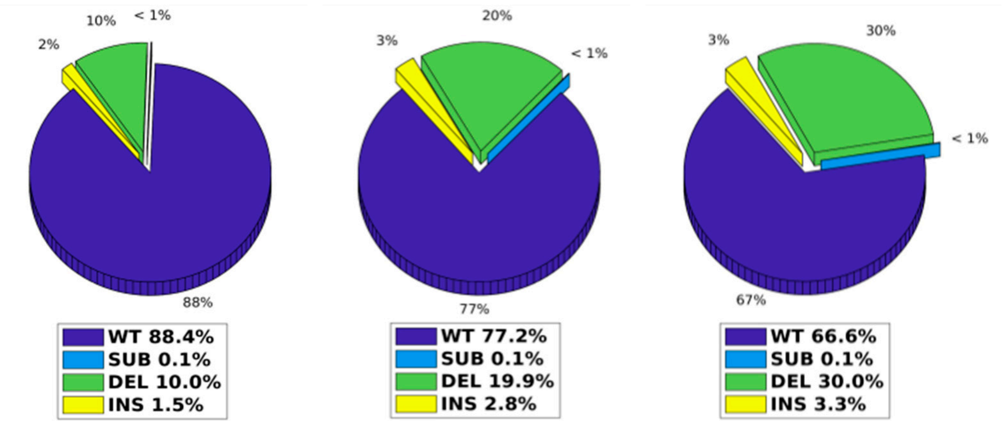

(**B**) Stop-light HEK293T cells – HPRT gRNA – 25, 50, and 100 ng Cas9/well

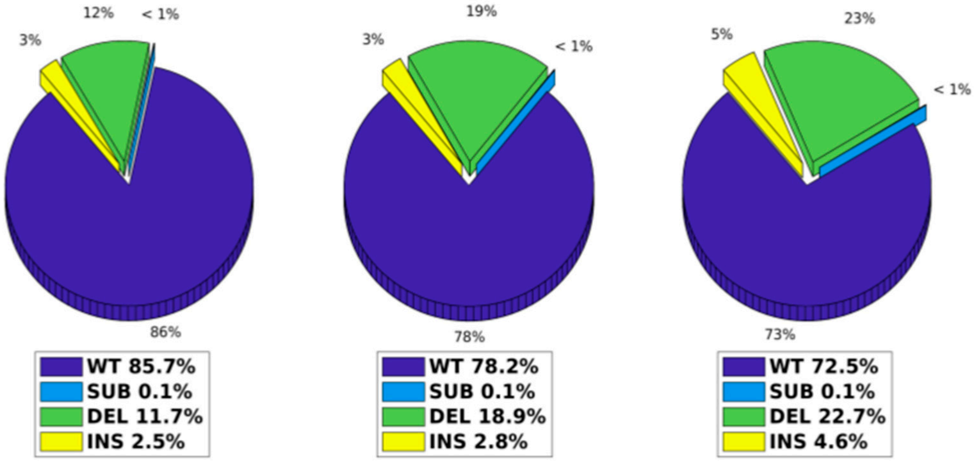

(C) HEK293T cells – HPRT gRNA – 25, 50 and 50 ng Cas9/well

Figure S6. (**A, B & C**) Shows the distribution of indel variants in SL and WT cells treated with an increasing amount of RNP-PF14 targeting the SL construct or the HPRT gene. SUB = substitution, DEL = deletion, INS = insertion. The pie charts were generated by TigerQ AB, Lund, Sweden.
